# Challenging cases in infection prevention and control: proceedings from SHEA Spring 2025

**DOI:** 10.1017/ash.2025.10286

**Published:** 2026-02-09

**Authors:** Jessica Seidelman, Tsun Sheng N. Ku, Anna LaFountain, Curtis J. Donskey, Edoabasi McGee, Rachel Pryor

**Affiliations:** 1 Infectious Diseases, Duke University Hospital, Durham, USA; 2 Rocky Vista University College of Osteopathic Medicine, Billings, MT, USA; 3 Billings Clinic, Billings, USA; 4 Case Western Reserve University School of Medicine, Cleveland, OH, USA; 5 Philadelphia College of Osteopathic Medicine, Philadelphia, PA, USA; 6 Virginia Hospital Center Arlington Health System, Arlington, VA, USA

## Abstract

Hospital epidemiologists and infection prevention professionals are frequently required to make high-stakes decisions in complex clinical scenarios where evidence-based guidance is limited or absent. These decisions often carry significant implications for patient safety, healthcare worker protection, hospital operations, and legal or financial risk. This article presents three challenging cases originally featured during the “Interesting Cases” session at the Society for Healthcare Epidemiology of America (SHEA) Spring 2025 Conference, with the goal of sharing practical lessons learned and contributing to the evolving literature in healthcare epidemiology. The first case describes the intraoperative contamination of a polyethylene liner during emergency revision total hip arthroplasty, highlighting limited data guiding implant salvage and the role of antiseptic decontamination, interdisciplinary communication, and institutional preparedness. The second case examines infection prevention risks associated with a temporary hospital heating, ventilation, and air conditioning (HVAC) shutdown during a COVID-19 surge, emphasizing the use of real-time ventilation assessment tools such as carbon dioxide monitoring to guide mitigation strategies. The third case details the application of failure mode and effect analysis (FMEA) to develop an infection prevention and control policy for the educational use of non-transplant cadaveric tissue in patient care areas—an area with no existing guidelines. Collectively, these cases illustrate the realities of decision-making under uncertainty in hospital epidemiology and demonstrate how structured risk assessment, proactive planning, and cross-disciplinary collaboration can mitigate potential harm. Sharing these experiences provides practical insights and reinforces that, even in the absence of definitive guidance, systematic approaches can support safe infection prevention decision-making.

## Introduction

The work of hospital epidemiologists and infection preventionists is inherently varied and consequential. Their decisions can have infectious, operational, and/or financial implications for the entire hospital, affecting patients, visitors, and healthcare workers alike. The work may require making complex decisions independently, with little or no expertise from others within the facility. These decisions can have unintended impacts and often must be made during time-limited scenarios and/or based on insufficient evidence. Navigating these factors while making critical decisions can cause considerable stress and difficulty.

This article presents three cases where hospital epidemiologists made critical decisions with limited evidentiary data. Originally shared during the “Interesting Cases” session at the Society for Healthcare Epidemiology of America’s (SHEA) Spring Conference in April 2025, the session presenters have been invited to share their cases with ASHE readers to communicate lessons learned and add to the limited body of literature available during each clinical situation. This type of session has been a part of the SHEA Spring Conference since 2017.

Though readers may never encounter one of these exact cases, the authors believe that providing their experiences, decision-making processes, and outcomes will be valuable to readers. Although the decision-making duties in hospital epidemiology can be substantial, these cases illustrate that such challenges are widely shared and that practitioners need not confront them in isolation.

## Case 1. A dropped liner and a difficult decision

Presented by: Jessica Seidelman MD, MPH

### The problem: an implant accidentally dropped on the operating room floor

During a revision total hip arthroplasty (THA) for a 94-year-old woman with a periprosthetic fracture, the surgical team encountered a critical intraoperative dilemma: the polyethylene (PE) liner was accidentally dropped on the operating room (OR) floor. With no replacement liners available and the patient’s vital signs becoming tenuous under anesthesia, the team faced a high-stakes decision. Should they proceed using the contaminated implant, attempt disinfection, or abort the procedure? This situation raised a timely and important question for infection prevention clinicians: is there a safe, evidence-based path forward when sterile surgical implants that cannot undergo flash sterilization are contaminated mid-procedure?

### Data review: what the literature tells us about dropped implants

While the so-called “five-second rule” may be debunked in food safety, a similar logic persists in high-pressure surgical environments. Approximately 61.5% of implant drops occur during emergency surgeries—precisely the kind of unpredictable and stressful scenario faced in this case.^1^ Despite this frequency, clear, universally adopted guidelines for managing dropped implants remain elusive.

Studies of OR contamination reveal that surfaces are frequently colonized by pathogens, making any dropped item—particularly an implant—potentially dangerous. Data from Weber and colleagues, among others, underscore the real risk posed by contaminated OR environments.^2–4^

A 2019 literature review by Vautrin et al.^5^ explored expert responses to dropped PE implants. They found that more than half of experienced orthopedic surgeons would prefer to delay definitive implantation rather than use a contaminated liner. Other strategies included soaking the implant in antiseptic solutions or temporarily using a provisional implant.^5^

But what antiseptic solution, if any, is effective? A key study comparing 0.5% chlorhexidine gluconate (CHG), 10% povidone-iodine (PI), and 70% ethanol for decontaminating dropped bone grafts found that CHG was far superior in reducing contamination (5.4% positive cultures) compared to PI (67.6%) and ethanol (81.1%).^6^ These findings support the use of CHG as the antiseptic of choice when attempting salvage.

### The solution & infection prevention takeaways: navigating risk with evidence and communication

In this case, the team opted to soak the PE liner in a sterile 4% CHG plus alcohol solution for 10 minutes before re-implanting it. The patient’s family and the hospital’s legal and risk teams were informed. Notably, no additional antibiotics were administered postclosure. Nearly a year later, the liner remains in situ with no sign of infection—a fortunate outcome in a high-risk scenario.

This case reinforces several key infection control lessons:

1. **Stock Adequately**: Hospitals must ensure that sufficient backup implants and liners are available for all major procedures, especially revisions and emergencies.
2. **Establish Protocols**: Institutions should develop and implement standardized protocols for decontamination and decision-making when sterile field breaches occur. While the 4% CHG plus alcohol was used in this specific instance, institutional protocols may vary based on available evidence and agent compatibility with materials.
3. **Train Regularly**: Regular IPC training for surgical staff can improve preparedness and reduce risky improvisations during intraoperative crises.
4. **Prioritize Collaboration**: Clear communication between surgical teams, anesthesiologists, infection control professionals, and hospital leadership is critical in navigating these rare but consequential situations.

**Figure 1.**
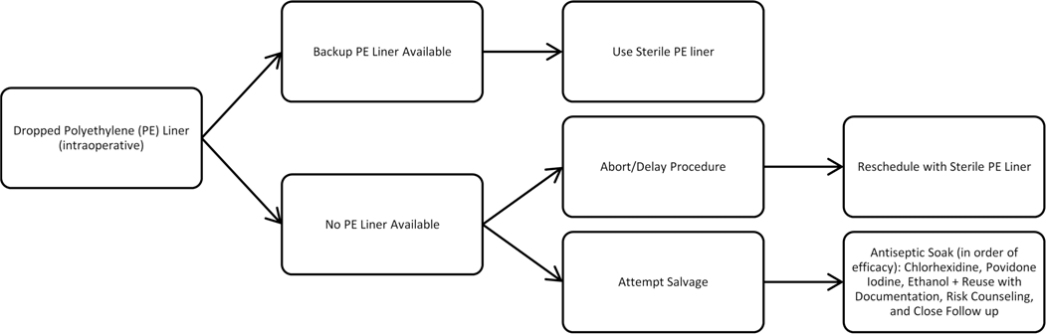
Decision algorithm for management of an intraoperatively dropped polyethylene (PE) liner.

Figure 1 outlines recommended steps when a PE liner becomes contaminated, including use of a sterile backup liner when available and salvage or rescheduling strategies when no replacement is present. Ultimately, while some level of contamination in the OR may be inevitable, its impact can be mitigated with planning, evidence-based action, and cross-disciplinary teamwork. The “10-second rule” doesn’t apply in surgery—but with the right safeguards in place, even a dropped implant doesn’t have to spell disaster.

## Case 2. A plan of attack when the HVAC is out of whack

Presented by Curtis J. Donskey MD

### The problem: temporary shutdown of the HVAC system supplying half the hospital

In an acute care facility, the hospital epidemiologist was called into an urgent meeting to discuss the level of risk associated with a temporary shutdown of the heating, ventilation, and air conditioning (HVAC) system. The hospital has 2 central air-handling units, each providing ventilation to half the building. To complete essential system maintenance, 1 unit would have to be completely shut down for ∼8 hours. The shutdown would occur in the setting of a coronavirus disease 2019 (COVID-19) surge.

The HVAC shutdown raised several practical questions for the infection prevention program. Will the non-ventilated side of the building have stagnant air, or will there be substantial flow of air from the ventilated half of the building? Are there tools that can be used to monitor ventilation and air quality during the shutdown? Are there interventions that can be used to minimize the risk to staff and patients?

### Data review: little information is available on the impact of HVAC shutdowns

HVAC systems regulate indoor temperature and humidity and provide adequate ventilation to control odors and reduce risk for transmission of airborne pathogens.^7,8^ Little information is available on the infection risks associated with temporary HVAC shutdowns.^7^ However, airborne infectious disease outbreaks have occurred when HVAC systems have been poorly maintained.^7^ Therefore, during temporary shutdowns, measures such as use of portable high efficiency particulate air (HEPA) cleaners, relocation of immunocompromised patients, and monitoring of ventilation have been recommended.^7^

### Tools to monitor ventilation: you can’t improve what you don’t measure

During the COVID-19 pandemic, our infection prevention program gained experience in the use of practical tools to assess ventilation (eg, carbon dioxide monitoring, measurement of clearance of released aerosol particles) and airflow (eg, condensed moisture or smoke).^9–11^ These tools can be useful to identify measures that are likely reduce viral transmission versus those that may amount to “hygiene theater” or even be counterproductive. Carbon dioxide monitoring with handheld devices may be particularly useful because it is inexpensive, easy-to-use, and provides immediate results.^9,10^ Carbon dioxide levels rise in indoor areas that are inadequately ventilated because the concentration of carbon dioxide in exhaled breath is ∼40,000 parts per million (ppm) versus ∼450 ppm in outdoor air; levels above 800 ppm are considered an indicator of suboptimal ventilation for the number of people present.^9^ The most important limitation of carbon dioxide monitoring is that it does not account for filtering of air (eg, portable HEPA air cleaners).^9^

### Impact of the HVAC shutdown

We used carbon dioxide monitoring as a primary tool to assess ventilation during the HVAC shutdown. A detailed description of this assessment which also included measurement of clearance of aerosolized particles has been reported previously.^12^ The negative-pressure rooms (N = 15), bathrooms, and soiled utility rooms remained operational on the side of the hospital with the air handler shutdown because they have dedicated exhaust systems.^7^ We hypothesized that operation of these exhaust systems might provide sufficient negative pressure to facilitate movement of air from the ventilated to the non-ventilated side of the building.

**Figure 2.**
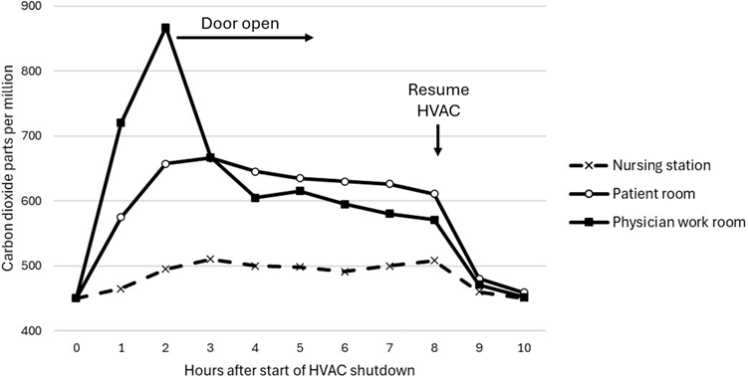
Carbon dioxide concentrations in typical nursing station, patient room with the door open, and physician work room during and after temporary shutdown of the heating, ventilation, and air conditioning (HVAC) system supplying half of the hospital.

Figure 2 shows carbon dioxide levels during and after the shutdown in a nursing station, patient room, and physician work room that are typical of findings for multiple assessments on 4 floors of the hospital. Carbon dioxide levels increased only to a modest degree (∼50 ppm) in the nursing stations during the shutdown. This finding was consistent with the observation that people in the hallways could feel currents of airflow moving from the ventilated side of the building to the non-ventilated wards. Carbon dioxide levels increased to a greater degree in patient rooms with the doors open but remained below 800 ppm. In physician team workrooms, carbon dioxide levels rose above 800 ppm when 5 to 6 people were present with the door closed, with a rapid decrease when the door was kept open.

### Infection prevention lessons learned

Our investigation resulted in several lessons learned. First, carbon dioxide monitoring can be used to provide a real-time assessment of the impact of HVAC shutdowns and of interventions undertaken to improve ventilation. Second, the shutdown of 1 air handler in a hospital may have a relatively modest impact on ventilation if other components of the ventilation system continue to function (eg, additional air handlers, dedicated exhaust systems for negative-pressure rooms and bathrooms). Third, practical interventions such as opening doors to occupied rooms may facilitate air exchange when the vents supplying the room are not functioning. Although our findings provide some reassurance, we would encourage other Infection Prevention programs to conduct and report similar evaluations during HVAC shutdowns.

## Case 3. The application of the failure mode and effect analysis in the development of an infection prevention and control policy for the educational use of non-transplant cadaveric tissue in patient care settings

Presented by Tsun Sheng N. Ku MD

### The problem: how do we safely allow surgeons to practice on cadavers in a surgical suite?

Infection prevention and control (IPC) practices at many healthcare institutions are usually based on published guidelines and recommendations, such as those from the Centers for Disease Control and Prevention, SHEA, and Association for Professionals in Infection Control, which primarily focus on common scenarios in healthcare settings. Occasionally, hospital infection prevention teams (IPTs) are asked to develop IPC protocols for scenarios that are not included in those guidelines. Developing such protocols often requires knowledge of the infectious diseases risks associated with those scenarios. For some scenarios, however, those risks are not readily apparent.

The IPT at Billings Clinic was recently asked to develop an IPC protocol that would permit the use of fresh frozen non-transplant cadaveric tissue (FFNTCT) in a surgical suite for an educational demonstration of a new implantable orthopedic device. Although no guidelines were available to develop a protocol for this scenario, our infection preventionists recognized potential risks to both the medical staff and patients. As such, we describe here the use of failure mode and effect analysis (FMEA) as a technique to assess potential risks for this activity and develop strategies to mitigate those risks.

### Data review: nothing has been published about how to have cadavers for demonstrations in surgical suites

FFNTCT is usually procured from non-transplant tissue banks (NTTBs) that sell or lease human bodies and body parts for medical education and research, which receive bodies through donations.^13^ They reportedly screen the donations for infectious diseases and may certify that they are pathogen-free. NTTBs, however, are not tightly regulated. On the other hand, NTTBs accredited by the American Association of Tissue Banks follow standards for screening donations for pathogens and certifying them as pathogen-free.

Our team’s first step was to perform a literature search for IPC case reports involving FFNTCT in healthcare settings. No publications were found, which led our team to post in the discussion forums of peer networks for comments about this scenario. The responses were primarily opinions, ranging from suggestions of not allowing the practice to one response supporting the practice, as the respondent (an orthopedic surgeon) felt the benefits outweighed any potential risks. However, none of the respondents indicated having any experience with this scenario.

The team then interviewed the device vendor representative about the demonstration process. The representative indicated that these demonstrations are typically performed at the company’s site. Occasionally, the vendor arranges a demonstration at a local venue, but not at a patient care site. The vendor provides FFNTCT and arranges for its delivery and removal. However, the vendor did not have a specific IPC protocol for local demonstrations.

### The solution: building an IPC policy from scratch

Billings Clinic has a quality improvement team whose members are certified in Lean Six Sigma and the Kaizen process. A member of this team was assigned to work with the IPT on this project. After discussing the risk analysis challenges, the FMEA was proposed to be the preferred risk analysis tool for the project.

FMEA is a prospective risk analysis tool designed to help predict vulnerabilities within a system process.^14^ It has been used in engineering, manufacturing, and the military for decades. However, there has been a recent increase in interest in its use in healthcare settings, especially for patient safety and performance improvement initiatives.^15,16^ A modified version of FMEA exists that is specific to healthcare (ie, HFMEA), which uses a hazard scoring matrix instead of a risk priority number to help prioritize the risk of potential failures.^17^

**Figure 3.**
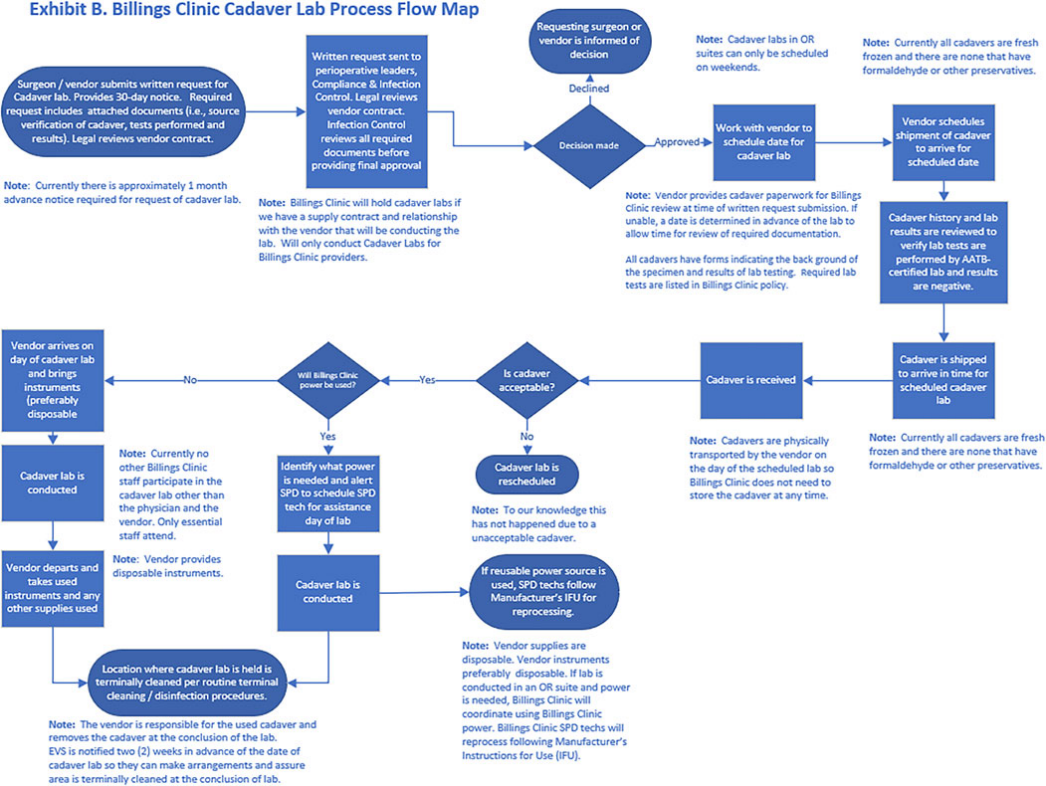
Billings clinic cadaver lab process flow map.

The team first developed a step-by-step process leading to and following the device demonstration (Figure 3). Each step was assessed for potential failures (Figure 2). Although the primary focus of the FMEA was on IPC and disinfection, other factors were considered, including potential disruptions of concurrent patient care activities and exposure of the FFNTCT to individuals not associated with the activity.

**Figure 4.**
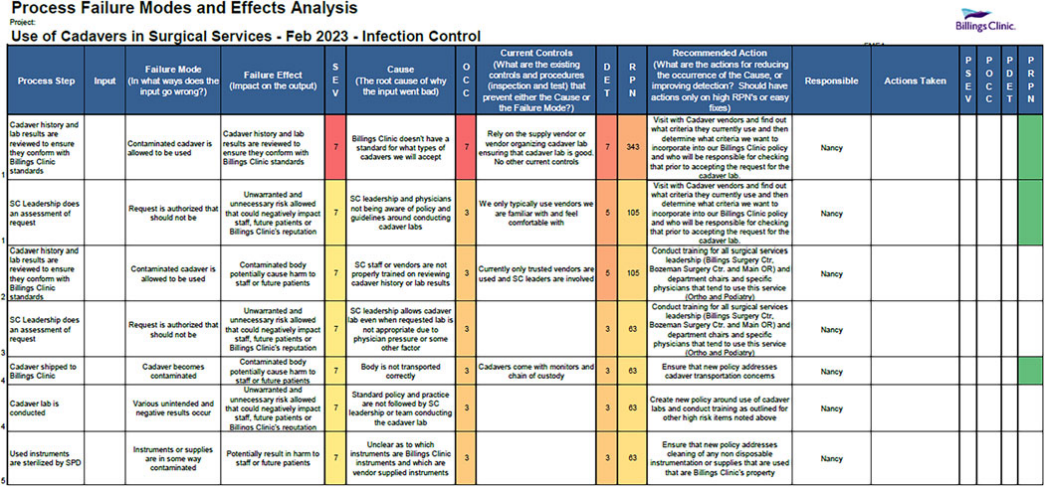
Process failure modes and effects analysis for use of cadavers in surgical services.

A list of potential failures with high probability and severity was then compiled, and mitigating actions were proposed for each failure (Figure 4). The actions were distributed to the appropriate stakeholders (eg, surgery and perioperative leadership, vendor representative, environmental services, legal, compliance, etc.) for review. Our IPT then drafted the policy, outlining the processes for proposing, approving, and implementing product demonstrations involving cadaveric tissue in patient care areas.

Although the product demonstration was cancelled before the policy was approved, Billings Clinic was able to implement this policy for another demonstration. The process had performed as expected. Thus far, no adverse outcomes have been identified.

### Infection prevention lessons learned

FMEA appears to be a useful tool for developing IPC protocols for atypical situations. Furthermore, it can also be used to analyze existing IPC processes for failures for optimizations.^16^ As healthcare systems become more complex, mitigating potential process failures becomes more important. Thus, hospital IPTs should include FMEA as a tool for assessing their IPC measures.

## Conclusion

The various implications highlighted throughout the three cases presented show the reality of hospital epidemiologists’ challenges that do not always conform to neatly published guidelines. Navigating uncertainty in scenarios ranging from a dropped implant during emergency surgery to managing facility-wide HVAC disruptions during a pandemic surge, or evaluating a new educational activity involving cadaveric tissue, are high-stakes decisions, and evidence-based guidelines do not completely address how to make these decisions. These three “Interesting Cases” contribute to the literature, enabling hospital epidemiologists to have more evidence, albeit in the form of case reports, to help navigate potentially similar cases. Fundamentally, the lesson these three cases share is that when faced with unprecedented scenarios, structured approaches like failure mode and effect analysis can transform uncertainty into manageable risk. All three cases represent systemic vulnerabilities (supply chain, infrastructure, novel procedures) where preemptive policy and risk assessment can build resilience Hospital epidemiologists must move beyond reactive problem-solving to proactive planning, ensuring adequate backup supplies, preestablished protocols for everyday emergencies, and regular cross-disciplinary training. When guidelines are absent, hospital epidemiologists can develop systematic risk assessment methodologies within a transparent and reproducible framework for policy development, which can be adapted across diverse scenarios. Ultimately, excellence in hospital epidemiology requires a balance of scientific rigor, practical creativity, and collaborative spirit—qualities exemplified in each of these challenging cases.

## References

[1] Khan SA , Kumar A , Varshney MK , Trikha V , Yadav C. Accidentally falling instruments during orthopaedic surgery: time to wake up! ANZ J Surg 2008;78:794–795. 18844911 10.1111/j.1445-2197.2008.04652.x

[2] Weber DJ , Rutala WA , Miller MB , Huslage K , Sickbert-Bennett E. Role of hospital surfaces in the transmission of emerging health care-associated pathogens: norovirus, *Clostridium difficile*, and *Acinetobacter* species. Am J Infect Control 2010;38:S25–S33. 20569853 10.1016/j.ajic.2010.04.196

[3] Yezli S , Barbut F , Otter JA. Surface contamination in operating rooms: a risk for transmission of pathogens? Surg Infect (Larchmt) 2014;15:694–699. 25317716 10.1089/sur.2014.011

[4] Weber DJ , Anderson D , Rutala WA. The role of the surface environment in healthcare-associated infections. Curr Opin Infect Dis 2013;26:338–344. 23743816 10.1097/QCO.0b013e3283630f04

[5] Vautrin M , Moerenhout K , Udin G , Borens O. Perioperative contamination of orthopaedic polyethylene implants, targeting devices and arthroscopes: experts decision tree and literature review. J Bone Jt Infect 2019;4:65–71. 31011510 10.7150/jbji.30613 PMC6470652

[6] Mat-Salleh MF , Sadagatullah AN , Ibrahim MY , et al. Are dropped bone grafts safe to be re-used? - An experimental study comparing efficacy of chlorhexidine, povidone-iodine and alcohol. Malays Orthop J 2021;15:70–76. 34429825 10.5704/MOJ.2107.011 PMC8381681

[7] Centers for Disease Control and Prevention. Guidelines for environmental infection control in health-care facilities. Background C. Air. https://www.cdc.gov/infectioncontrol/guidelines/environmental/background/air.html. Accessed March 2, 2025.

[8] Ha W , Zabarsky TF , Eckstein EC et al. Use of carbon dioxide measurements to assess ventilation in an acute care hospital. Am J Infect Control 2022;50:229–232. 34848292 10.1016/j.ajic.2021.11.017 PMC8627286

[9] Cadnum JL , Donskey CJ. If you can’t measure it, you can’t improve it: practical tools to assess ventilation and airflow patterns to reduce the risk for transmission of severe acute respiratory syndrome coronavirus 2 and other airborne pathogens. Infect Control Hosp Epidemiol 2022;43:915–917. 35379373 10.1017/ice.2022.103 PMC9021581

[10] Donskey CJ. High technology and low technology measures to reduce risk of SARS-CoV-2 transmission. Am J Infect Control 2023;51:A126–A133. 37890942 10.1016/j.ajic.2023.03.007

[11] Cadnum JL , Jencson AL , Alhmidi H , Zabarsky TF , Donskey CJ. Airflow patterns in double-occupancy patient rooms may contribute to roommate-to-roommate transmission of severe acute respiratory syndrome coronavirus 2. Clin Infect Dis 2022;75:2128–2134. 35476020 10.1093/cid/ciac334 PMC9129113

[12] Cadnum JL , Memic S , Eckstein EC , Donskey CJ. Evaluation of ventilation during partial shutdown of a hospital heating, ventilation, and air conditioning system for maintenance. Infect Control Hosp Epidemiol 2023;44:2099–2100. 37528772 10.1017/ice.2023.166

[13] The Body Trade: Cashing in on the Donated Dead. A *Reuters* series. Reuters. 2018. http://www.reuters.com/investigates/section/usa-bodies/. Accessed August 25, 2025.

[14] Stamatis D. Failure Mode and Effect Analysis: FMEA from Theory to Execution. Milwaukee, WI: ASQ Quality Press; 2003.

[15] DeRosier J , Stalhandske E , Bagian JP , Nudell T. Using health care failure mode and effect analysis: the VA National Center for Patient Safety’s prospective risk analysis system. Jt Comm J Qual Improv 2002;28:248–267. 12053459 10.1016/s1070-3241(02)28025-6

[16] Dawson A. A practical guide to performance improvement: failure mode and effects analysis. AORN J 2019;110:282–287. 31465564 10.1002/aorn.12780

[17] VHA National Center for Patient Safety (NCPS). Healthcare failure modes and effects analysis (HFMEA) guidebook. 2021. https://www.patientsafety.va.gov/professionals/onthejob/hfmea.asp. Accessed January 9, 2025.

