# Challenging cases in infection prevention and control: proceedings from SHEA Spring 2025 – CORRIGENDUM

**DOI:** 10.1017/ash.2026.10371

**Published:** 2026-04-01

**Authors:** Jessica Seidelman, Tsun Sheng N. Ku, Anna LaFountain, Curtis J. Donskey, Edoabasi McGee, Rachel Pryor

The author’s name was incorrectly spelled in the article as originally published (Anna LaFountaine). This has been corrected to Anna LaFountain in the HTML and PDF versions of the article.
